# Transactions of the State Medical Society of the State of New York

**Published:** 1855-08

**Authors:** 


Art. VI.—*Transactions of the State Medical Society of the State of New York.* Transmitted to the Legislature, Feb. 13, 1855.

---

The Transactions of the medical societies of the several States have grown to be an interesting and important department of our medical literature. We always take them up with the expectation of finding useful suggestions, histories of epidemics and cases that enlarge our knowledge, reports on medical progress, and new facts to aid the practitioner. The volume that comes annually from the Medical Society of the State of New York is always creditable to the professional industry and learning of that great commonwealth. The one before us contains a number of very able papers, the first of which is the annual address of the President, Prof. Coventry, on the "Philosophy of Medicine as a Science and an Art." We regret that we cannot find room for extracts from this excellent address.

The second paper is a report from the pen of Professor Frank. H. Hamilton, on Dislocations, with especial reference to their results—an elaborate and valuable paper. Every surgeon will be interested in reading the details which Dr. Hamilton has collected and arranged with great care and fidelity. The report presents the results of his experience and observation for a period of twenty years. As the report is itself an abstract of cases, it will be impossible for us to give any synopsis of his observations.

The next article is an address on the "Needs, Duties, and Privileges of the Medical Profession," by John McCall, M. D. We are surprised in such an address to meet with the vulgarism, 'gents,' for gentlemen. It occurs in the following paragraph, which, certainly, to say the least of it, is curious:

> And here, gents, let me caution you to keep down your wonder, for we, too, have an ample supply thereof, while I assure you that it is now just fourteen years this month since I have held a patient by the arm, or abstracted blood, in any way, except in a few instances by cupping and leeching. The good people of Utica have no blood to lose, except in the service of their country; and I trust no such occasion will occur in my day.

The next papers contain an account of improved forceps for hare-lip operation; injury of the shoulder; report on the epidemics of the sixth districts; epidemics; Dr. Parkhurst's case of extra-uterine conception, the latter of which we transcribe in part:

No unusual symptoms attended her pregnancy; her catamenia ceased, quickening was felt at the usual time, and the motions of the child increased were observed during the usual period of pregnancy. At the expiration of eight and a half months, she had severe labor pains, following a sudden fright from the falling of a vessel into the fire while she was engaged in cooking. Her physician, Dr. Farewell, of Litchfield, was called; the labor pains continued for several hours with regularity and force, but at length subsided and she remained comfortable for two or three weeks. Her health then began to decline, and the full period of pregnancy having passed by, her friends became extremely anxious, and availed themselves of the advice of Drs. Gulteau, Hull, Coventry, White and others. For a considerable time she was confined to her bed, and after a year and half of extreme suffering, her health began to improve, and was finally restored; during the remainder of her life she had good general health, but suffered occasionally from severe attacks of pain in the abdomen, which resembled labor pains. After her health was restored her catamenia returned and continued till the age of forty-five. She traveled much about the country and consulted various medical men, among others the late Prof. Willoughby, of Fairfield Medical College; her health continued remarkably good up to the time of her death, and at the age of seventy-six she was accustomed to walk five miles from her residence to the village and back again.

The specimen with its covering cyst weighed eight pounds at the time of its removal. The external surface of the envelope was smooth and white, composed of concentric layers of fibro-cartilage varying at different points, from a line or two to three-fourths of an inch in thickness. It had no connections with the abdominal viscera or walls, but was slightly attached to the fallopian tubes and omentum. The external surface of the fœtus, was encrusted with earthy substance, of sufficient thickness to preserve its form when dried. The interior seems to be a soft substance resembling adipocire.

Next follows an account of a fatal operation for the removal of a tumor from the neck, which surgeons will read with great interest.

To this succeed two biographical sketches, one of Prof. James Webster, the other of Dr. Daniel Ayres. These are followed by an elaborate history of epidemic cholera at Troy, in 1854, by Drs. Brinsmade and Seymour, from which we copy the following:

*Is Cholera Contagious?*—The only cases bearing upon this subject, unless some already narrated may be considered as relating to this question, are case 380, rear of 171 Second st., Troy—a woman who had been no nearer any infected district than her residence, but had been attending case 278, which case had contracted the disease in attending cases 253–4, corner of Third st. and the nail factory road. Also case 271, corner of Grand Division and Maiden lane. This man visited in the evening a friend of his, case 263, at No. 5 North Fourth street, and spent two or three hours in rubbing and assisting him while in a collapse. He returned home and went into a collapse himself in an hour or two, and was dead before morning. The father of case 56 came from the country and took her and her husband home with him, and then sickened and died. The wife of case 87, after the death of her husband, went into the country to the house of her relatives. She was taken sick there herself, and a brother-in-law in the same house became sick. Both recovered. They ascribed the case of the latter to fright. These are the only cases I now think of, bearing upon the case of contagion. It will be observed that all except the last case either resided here or had been here; of course they were all, independent of the immediate exposure, more or less under the influence of the cholera poison. I commenced the season strongly disposed to believe in the contagious character of the disease, but a careful consideration of the cases bearing on this question satisfies me that there are none which cannot be explained by the influence which excitement, fatigue and fear undoubtedly have in bringing the subject of them within reach of the cholera poison already acting on them. The most that they can be claimed to prove is that isolated cases intensify the action of the poison in localitities already more or less under its influence. I know of no cases which clearly prove the communicability of the disease in an unquestionably healthy locality.

*Is the disease always preceded by diarrhœa?*—Some of the cases already mentioned are a sufficient answer to this question. There are occasional cases occurring in which it is indisputable that no diarrhœa or even vomiting exists—not only prior to the full development of the attack, but up to the fatal termination. There can be no doubt, however, that the vast majority of what I call *initial* cases are preceded by a diarrhœa which affords sufficient time for arresting the disease; but I very much fear that a careful investigation and an analysis of the cases occurring here and elsewhere would show that there is quite a respectable number of cases which may be called secondary, *i. e.*, depending on the initial, and which would not have occurred without them, which are not preceded by diarrhœa, or if preceded by it, the diarrhœa, the partial collapse and the pulseless stage follow each other so rapidly as to make but little difference in whether treatment is commenced in one stage or the other.

The concluding article of the volume is by Dr. Green, and is devoted to an inquiry into the efficacy of injections into the bronchial tubes, but as we have, in another place, discussed this subject at considerable length, we need not enter into it here.

